# Knowledge Distillation Facilitates the Lightweight and Efficient Plant Diseases Detection Model

**DOI:** 10.34133/plantphenomics.0062

**Published:** 2023-06-28

**Authors:** Qianding Huang, Xingcai Wu, Qi Wang, Xinyu Dong, Yongbin Qin, Xue Wu, Yangyang Gao, Gefei Hao

**Affiliations:** ^1^ State Key Laboratory of Public Big Data, College of Computer Science and Technology, Guizhou University, Guiyang 550025, China.; ^2^ Text Computing & Cognitive Intelligence Engineering Research Center of National Education Ministry, Guizhou University, Guiyang 550025, China.; ^3^ National Key Laboratory of Green Pesticide, Guizhou University, Guiyang 550025, China.

## Abstract

Plant disease diagnosis in time can inhibit the spread of the disease and prevent a large-scale drop in production, which benefits food production. Object detection-based plant disease diagnosis methods have attracted widespread attention due to their accuracy in classifying and locating diseases. However, existing methods are still limited to single crop disease diagnosis. More importantly, the existing model has a large number of parameters, which is not conducive to deploying it to agricultural mobile devices. Nonetheless, reducing the number of model parameters tends to cause a decrease in model accuracy. To solve these problems, we propose a plant disease detection method based on knowledge distillation to achieve a lightweight and efficient diagnosis of multiple diseases across multiple crops. In detail, we design 2 strategies to build 4 different lightweight models as student models: the YOLOR-Light-v1, YOLOR-Light-v2, Mobile-YOLOR-v1, and Mobile-YOLOR-v2 models, and adopt the YOLOR model as the teacher model. We develop a multistage knowledge distillation method to improve lightweight model performance, achieving 60.4% *mAP*@ .5 in the PlantDoc dataset with small model parameters, outperforming existing methods. Overall, the multistage knowledge distillation technique can make the model lighter while maintaining high accuracy. Not only that, the technique can be extended to other tasks, such as image classification and image segmentation, to obtain automated plant disease diagnostic models with a wider range of lightweight applicability in smart agriculture. Our code is available at https://github.com/QDH/MSKD.

## Introduction

Plant diseases are a leading cause of reduced crop yields, as crops are susceptible to infection during growth, and treatment opportunities are often missed when clear disease symptoms emerge. Given that plants are the primary source of human food [1] and a critical raw material for the light industry, their reduced production could lead to increased food crises [2] and higher costs of production. Studies show that between 702 and 828 million people were hungry in 2021, with an anticipated 670 million people still facing the threat of hunger by 2030 [3]. While most plant diseases have effective treatment options, they still cause 20% to 40% of food losses annually [1]. As such, traditional treatment options for plant diseases are no longer the sole solution for improving crop yields, and it is imperative to explore timely plant disease diagnosis methods and effective disease spread control strategies [4].

Traditional plant disease diagnosis methods [4] mainly rely on professionals for disease diagnosis and have limited applicability. To free up the workforce, plant disease diagnosis methods based on digital image processing and traditional machine learning have attracted widespread attention [5], which extract and process disease features and perform classification to achieve disease diagnosis [6,7]. For example, Tete et al. [8] use threshold segmentation and the K-means clustering algorithm to separate diseased areas from plant leaf images, which are classified by a neural network to achieve disease identification. Griffel et al. [9] collect near-infrared and short-wave infrared wavelength data by remote sensing for training and then exploit a machine learning classifier to identify potatoes infected with Potato Virus Y. However, the above methods still exist 2 problems. On the one hand, they are only applicable to identify single plant diseases. On the other hand, plant disease features are difficult to collect and are easily lost. The effectiveness of these methods with insufficient generalization ability can be greatly reduced when the number of categories of identified diseases increases.

**Fig. 1.**
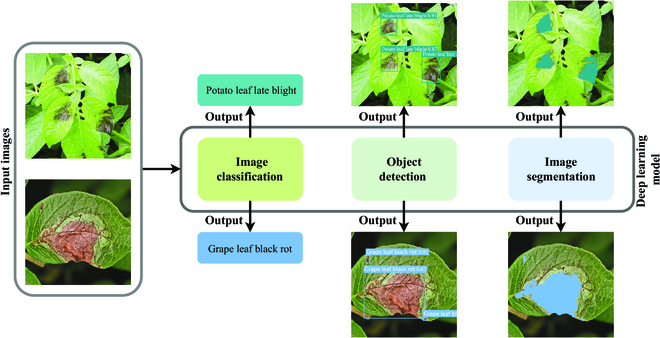
Examples of the plant disease diagnosis methods based on deep learning.

Recently, as deep learning has made substantial breakthroughs in image recognition, researchers have begun exploring how to use these techniques for plant disease diagnosis [10–13]. Currently, the plant disease diagnosis methods based on deep learning are mainly implemented through image classification, object detection, and image segmentation, as shown in Fig. 1. For example, Johnson et al. [14] propose an object detection model based on the Mask Region-based convolutional neural network (Mask R-CNN) [15], which is trained using images in RGB (red-green-blue) color space and images converted from RGB color space to other color spaces to achieve potato wilt identification. Bierman et al. [16] adopt a complementary metal oxide semiconductor sensor camera to capture images of grape leaves suffering from powdery mildew and exploit a convolutional neural network based on GoogleNet [17] to estimate the severity of the disease. Lin et al. [18] propose a semantic segmentation model for cucumber powdery mildew based on a convolutional neural network that segments the disease at the pixel level. Compared with plant disease diagnosis based on traditional machine learning, these aforementioned methods are considerably improved in applicability and transferability.

**Fig. 2.**
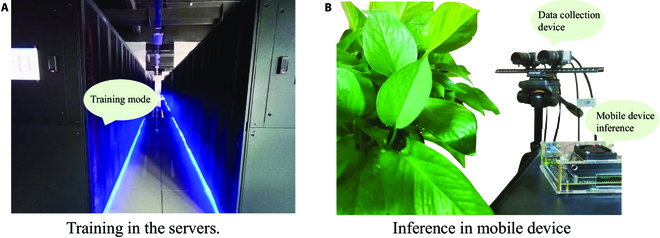
Comparison of model training and inference in reality. A lightweight model is extremely easily implemented on a mobile device than a heavy model, which can be used in smartagriculture, especially the edge device of the field. (A) Training in the servers. (B) Inference in mobile device.

However, the plant disease diagnosis methods based on deep learning increase accuracy at the risk of increasing computational load and decreasing detection speed, which leads to a restricted application in reality. We adopt the high-performance cluster computer for model training to obtain the best performance and attempt to deploy it to the terminal device that can be carried on the mobile side for inference and application, as shown in Fig. 2. Numerous studies are showing that through practice that the amount of parameters of the model trained on the high-performance cluster computer is too large and it is difficult to infer effectively on the terminal device [19–22]. Therefore, we need to explore more suitable methods for plant disease diagnosis by making a trade-off between the accuracy and speed of the model. Compared to computationally fast image classification methods, object detection methods can not only identify classes of plant diseases but also localize and count them [6,23]. It is noteworthy that image classification exhibits limitations in comprehending the semantic and contextual information of objects depicted in images. In contrast to image segmentation methods that support disease classification and localization, object detection methods have a definite advantage in terms of computational speed. However, existing plant disease diagnosis methods based on object detection are still limited to single crops or single-disease diagnosis [24–28]. Moreover, for the vast majority of general object detection models, there exists a negative correlation between accuracy and speed, whereas a positive correlation between accuracy and the number of parameters is observed [29–31]. This point drives us to explore more lightweight and accurate object detection-based plant disease diagnosis methods to achieve accurate diagnosis of multiple diseases across multiple crops in reality.

In our study, we propose a lightweight and efficient method via knowledge distillation for plant disease diagnosis based on object detection when facing multiple diseases across multiple crops. More specifically, we present 2 strategies for rapidly obtaining lightweight models and propose a multistage knowledge distillation method (MSKD) to enhance the performance of these models. The MSKD guides lightweight student models for learning through a complexly structured teacher model, in the backbone, neck, and head distillation stages. During the backbone and neck distillation stages, the focal and global distiller (FGD) [32] guides the student model using the middle layer features of the teacher model. Furthermore, we design a diversity knowledge transfer module for the plant disease category in the head distillation stage to drive the teacher model to guide the student model for plant disease category diversity learning. In the experiments, we evaluate our proposed methods in terms of performance, complexity, and computational requirements through a large number of experiments. Finally, we show that the proposed lightweight models can be easily deployed to mobile or embedded devices with limited computational power and high performance, such as field miniaturization equipment or unmanned aerial vehicles, to achieve efficient disease diagnosis of plants in large-scale agricultural fields at low cost.

## Materials and Methods

In this section, we introduce the MSKD in detail. Dataset describes the dataset required for the experiment. Multistage knowledge distillation describes the framework of MSKD. Student model explains the objective function of MSKD with loss terms. Object function reports the details of the evaluation methods for the experiments.

### Dataset

**Fig. 3.**
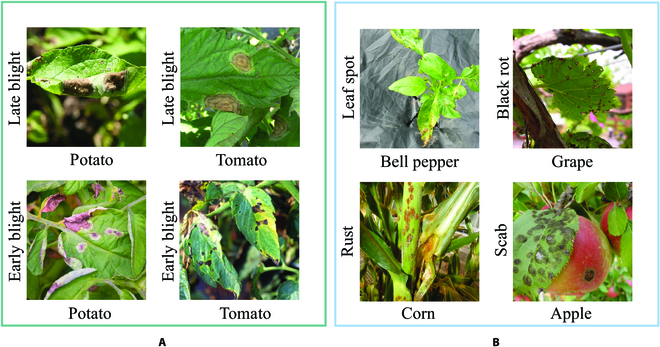
Examples of the relationship between multiple crops and multiple plant diseases.

Currently, there are thousands of different crops worldwide [33], including field crops, fruit trees, vegetables, ornamental crops, medicinal plants, forest trees, etc. As shown in Fig. 3A, one crop may be threatened by multiple plant diseases, and one plant disease may threaten multiple crops. Furthermore, different crops growing in the field are often threatened by the same or multiple plant diseases, as shown in Fig. 3B. Due to the difficulties in collecting high-quality data on multiple crops with multiple diseases, researchers have difficulty using deep learning methods with large parameter scales when exploring plant disease diagnosis methods.

**Fig. 4.**
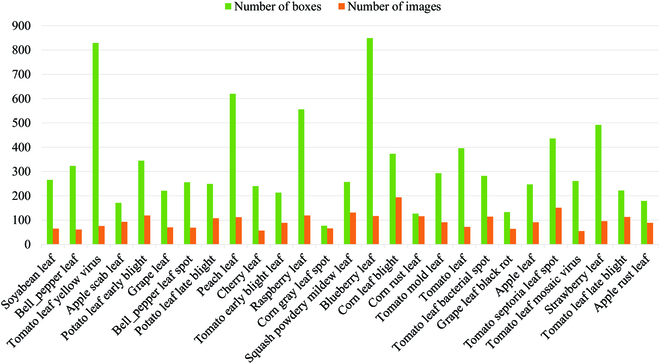
Number of images and bounding boxes for each category on PlantDoc [35] dataset.

In order to find more suitable plant disease datasets for our proposed method, we compare 4 plant disease datasets and introduce 2 general image object detection datasets as references. As shown in Table 1, some plant disease datasets have overly simple image backgrounds and do not provide object detection annotations, such as PlantVillage [34]. Other plant disease datasets can only be applied to diagnostic tasks for the single plant disease species, such as Potato Blight [14] and Citrus Disease [26], and the number of publicly available datasets is particularly limited. In contrast, the PlantDoc [35] dataset is a publicly available dataset that can be applied to diagnosis based on classification and detection tasks. In detail, this dataset includes images of multicrops with multidiseases in diverse backgrounds, which are suitable for our proposed approach, as shown in Fig. 4. However, compared to general image object detection datasets, such as PASCAL VOC [36] and COCO [37], the PlantDoc [35] dataset is far inferior to them in terms of the number of images, applications, and categories.

**Table 1.** Datasets comparison between plant disease diagnosis and image object detection.

Dataset	Pictures	Public available	Applications	Categories	Collection environment
PlantVillage [34]	50,000	TRUE	Classification	14 crop	Plain
				26 diseases	
Potato Blight [14]	1,840	FALSE	Detection	Potato blight	Complex
Citrus Disease [26]	2,684	FALSE	Detection	3 diseases	Plain background
PlantDoc [35]	2,598	TRUE	Classification	13 crops	Complex/plain
			**Detection**	17 diseases	
PASCAL VOC [36]	18,459	TRUE	Classification	20 categories	Complex/plain
			Detection		
			Segmentation		
COCO [37]	330,000	TRUE	Classification	81 Categories	Complex/plain
			Detection		
			Segmentation		

### Multistage knowledge distillation

**Fig. 5.**
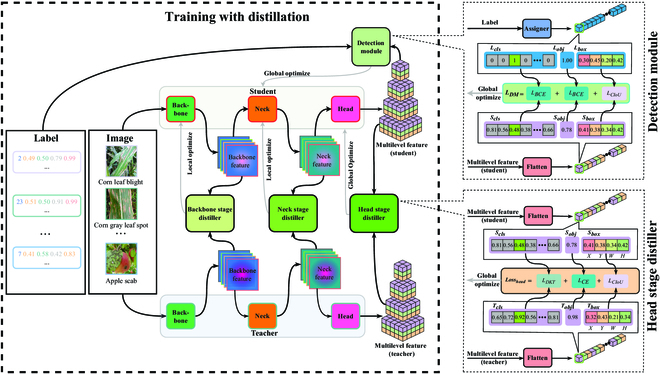
Framework of the MSKD. The input of both the teacher model and the student model are images, with the difference that the teacher model exploits the backbone and neck stage distillers to guide the backbone and neck parts of the student model for training. In particular, the head stage distiller provides feedback on the entire student model. The DM uses 3 loss functions to compare labels corresponding to images and multilevel student features and to provide feedback to the student model. Note that the details of the DM and head stage distiller are on the right side of the figure.

As shown in Fig. 5, we propose a MSKD for plant disease diagnosis based on object detection, which includes teacher and student models, backbone, neck, and head stage distillers, and a detection module (DM). More specifically, the given images are fed to the teacher and student models, and the teacher model instructs the student model on backbone and neck training using the backbone and neck stage distillers. In particular, the teacher model guides the entire student model through the head stage distiller for training. Throughout the training process, the teacher model uses the stand-by trained model and there are no training updates. To further train the student model, we introduce a DM that computes a loss function with the labels corresponding to the images and multilevel features from the student model, thus improving the model performance.

#### Teacher model

**Fig. 6.**
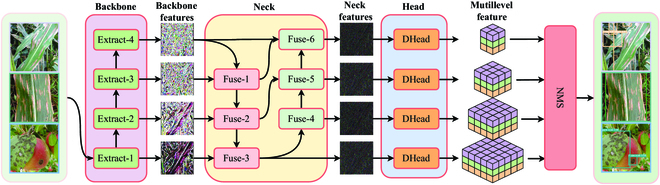
Architecture of the teacher model. The input image is converted to 4 backbone features with different semantic levels by 4 unique feature extraction stages of the backbone part, namely, extraction-1, extraction-2, extraction-3, and extraction-4, respectively. Then, the neck part uses the fusion modules to combine the backbone features to generate 4 neck features that encapsulate different levels of semantic information. Finally, the head part translates the neck features into multilevel features.

In the knowledge distillation method, the model that instructs other models to learn is called the teacher model, as opposed to the model that receives instruction from the teacher model, which is called the student model. Since the lesion area of plant images is mostly smaller than the background, plant diseases in the middle and late stages may have a more apparent lesion area than the initial stage. Therefore, we adopt the YOLOR [38] model as the teacher model, which consists of backbone, neck, and head parts, and incorporates implicit knowledge to improve the model performance, as shown in Fig. 6. In the teacher model, the backbone part references DarkNet [39], and the neck part is composed of the feature pyramid network (FPN) and the path aggregation network (PAN), while the head part consists of 4 YOLO detection heads [29] that support different scales.

In detail, the backbone consists of 4 feature extraction stages: Extract-1, Extract-2, Extract-3, and Extract-4. The neck contains 6 feature fusion stages, with the first 3 feature fusion stages (Fuse-1, Fuse-2, and Fuse-3) in YOLOR implemented through FPN, while the last 3 feature fusion stages (Fuse-4, Fuse-5, and Fuse-6) are implemented using PAN. The input image is converted to 4 backbone features with different semantic levels by 4 unique feature extraction stages of the backbone. In particular, the FPN enables the fusion of backbone features with different levels of semantics, allowing the YOLOR [38] model to efficiently combine high-level and low-level semantic information, while the PAN can effectively utilize low-level pinpointing information. After the backbone features have been fused, the neck part sends 4 neck features to the head part so that objects can be found at different scales. During the training stage, the head only translates the neck features into multilevel features. The multilevel feature representation contains multiple dimensions, each of which conveys a specific type of information. These dimensions can indicate the probability an object being present in a certain region of the image, the probability of that object belonging to a certain category, or the dimensions and location of the object if it is present in that region. It is worth mentioning that we refer to these 2 probabilities as confidence and category scores, respectively. However, during the inference stage, after the image has been processed by the backbone, neck, and head, the nonmaximum suppression (NMS) algorithm will be used to filter out potentially duplicated bounding boxes.

For the training of the teacher model, we adopt the pretrained model provided by Wang et al. [38] and fine-tune it on the PlantDoc [35] dataset. In addition, by using the k-means algorithm, we also get a set of width and height hyperparameters from the PlantDoc [35] dataset, as shown in Table 2. These hyperparameters are applied to the heads of both the teacher and student models to provide the initial value of object width and height for the head of the model, allowing the model to perform better. The effect of these hyperparameters will be demonstrated in impact of the initial values of the object boxes.

**Table 2.** Width and height applicable to plant diseases on PlantDoc [35] dataset.

No.	1	2	3	4	5	6	7	8	9	10	11	12
Width	58	120	100	200	155	300	209	227	399	572	398	597
Height	64	90	151	134	220	201	318	475	347	259	582	440

#### Multistage distillers

Most of the existing plant disease diagnosis models are based on supervised learning, and their accuracy is directly influenced by the supervised information in the dataset. However, the information provided by the labels of existing datasets is limited, and some models cannot fully exploit this limited information to achieve satisfactory results, especially lightweight models with simple structures. Knowledge distillation [40] is a method that can provide more useful information for model learning. For image classification models, different classification models output a vector of the same form when an image is inputted, and correspondence can be easily established between the outputs of different models. For object detection models, different numbers of bounding boxes may be output for the same image, and it is extremely difficult to establish correspondence between the outputs of these models. Therefore, some research [41,42] has focused on knowledge distillation methods for image classification models for plant disease diagnosis or other plant phenotype tasks. However, there is currently a lack of research on knowledge distillation methods for object detection models used in plant disease diagnosis. To address the above issues, we design backbone, neck, and head stage distillers to guide the student model using the teacher model for learning, as shown in Fig. 5.

**Fig. 7.**
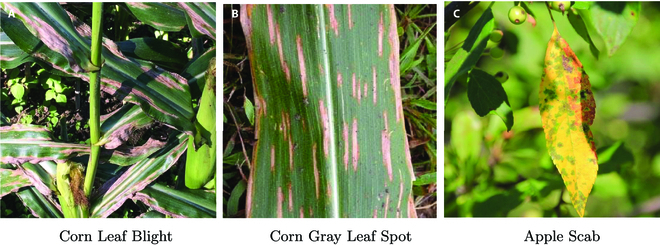
Examples of the commonalities and differences arising from the associations between different crop diseases. (A) Corn leaf blight. (B) Corn gray leaf spot. (C) Apple scab.

1. Backbone stage distiller. We have gotten the backbone stage distiller by changing the working position of the FGD [32]. This distiller employs a composite approach, comprising a neural network that captures both the global and local semantic information of the backbone features in both the teacher and student backbone sections, as well as a feature transformation function. In processing plant disease diagnosis, obtaining the key pixel points and the global relationships between pixel points is an important step. More specifically, key pixel points are the lesion areas that are focused on first when diagnosing diseases, while the global relationship between pixel points is the relationship between the lesion areas and their surroundings when performing disease category discrimination. The distiller uses the middle layer features of the teacher model’s backbone to guide the learning of the student model’s backbone, so that it not only provides the student model with global relationships between the pixel points acquired by the teacher model but also makes it focus on the key pixel points and channels of the teacher model. As illustrated in Fig. 5 the backbone stage distiller exclusively performs local optimization on the model, targeting solely the backbone part of the student model.

2. Neck stage distiller. Backbone feature fusion was completed for both the neck part of the teacher and student models, as shown in Fig. 6. The neck stage distiller uses the features provided by the neck of the teacher model to distill the neck of the student model, thus effectively guiding the student to a more appropriate fusion of features with different levels of semantic information. Notably, the neck stage distiller can indirectly optimize the backbone of the student model when optimizing the student model through back-propagation. In simple terms, the neck stage distiller locally optimizes both the neck and backbone parts of the student model, as shown Fig. 5. This distiller has the same structure as the backbone distillation stage.

3. Head stage distiller. In the natural environment, there may be commonalities and differences between different crop diseases. For example, corn leaf blight and corn gray leaf spot have a high degree of similarity in characteristics such as the distribution location and morphology of the diseases, as shown in Fig. 7A and B and . And then, we can find that there are disease differences between corn leaf blight and apple scab, but both diseases have the commonality of occurring on the leaves, as shown in Fig. 7A and C. Taking full advantage of these commonalities and differences can improve the detection performance of plant disease diagnostic methods. However, the existing plant disease detection datasets lack negative category information, which cannot result in idealized performance through entropy-based or mean square deviation-based loss functions.

To address the above issues, we design a diversity knowledge transfer module in the head stage distiller, which is mainly implemented through the *L_DTK_* loss function. This loss function calculates the discrepancy between the category scores *T_cls_* and *S_cls_* generated by the teacher and student models respectively, which can help the student model learn the relationship between categories from the proficient teacher model. The detailed depiction of the head stage distiller, illustrating this process, is presented in Fig. 5. It is worth mentioning that *L_DKT_* captures the diversity between negative categories and the diversity between positive and all negative categories. The effectiveness of this approach stems from the fact that all positive and negative category scores in the teacher model possess nonzero values. Since the output of the teacher model is similar to the real label, we employ the cross-entropy loss (*L_CE_*) to calculate the confidence difference between the *T_obj_* and *S_obj_* output by the teacher and student models, respectively, to provide additional guidance for the student model. Moreover, we apply *L_CIoU_* [43] to calculate the spatial information difference between *T_box_* and *S_box_* output by the teacher and student models, respectively. Thus, the output of the teacher model, which is similar to the real label, can be an effective guide for the student model. Thus, the overall loss function of the head stage distiller is as follows:

$$\mathrm{Los}s_{\mathrm{head}}=\omega_{1}\times L_{\mathrm{DTK}}\left(S_{\mathrm{cls}},T_{\mathrm{cls}}\right)+\omega_{2}\times L_{\mathrm{CE}}\left(S_{\mathrm{obj}},T_{\mathrm{obj}}\right)+\omega_{3}\times L_{\text{CIoU}}\left(S_{\mathrm{box}},T_{\mathrm{box}}\right),$$ (1)

where *ω*_1_, *ω*_2_, and *ω*_3_ are constants, *L_DTK_* and *L_CE_* are used to calculate cross-entropy loss, *S_cls_*, *S_obj_*, and *S_box_* represent the category scores, the confidence, and the spatial information of the object, respectively, output from the student model, while *T_cls_*, *T_obj_*, and *T_box_* are from the teacher model. In this equation, *L_BCE_* denotes binary cross-entropy loss function, and the computation method of *L_CIoU_* refers to reference [43].

### Student model

Our previous section details our research on enhancing model performance through external forces. In addition, we seek to improve the lightweight nature of the model from its own design. To this end, we propose 2 strategies for rapidly constructing lightweight models, and we utilize these strategies to construct 4 student models. The network architectures of the teacher and student models are similar, as shown in Fig. 6, with the difference that the student models are the YOLOR-Light-v1, YOLOR-Light-v2 model, Mobile-YOLOR-v1, and Mobile-YOLOR-v2 models obtained by optimizing the network structure details of the teacher model with 2 strategies. In addition, the class and number of convolutional layers for the teacher and student models are shown in Table 3.

**Table 3.** Infrastructure of the backbone and neck parts of the teacher and student models. † indicates that this is a special convolutional layer [55].

Model	1 × 1 Conv	3 × 3 Conv	Depthwise Conv †	Pointwise Conv †
YOLOR [38]	86	53	0	0
YOLOR-Light-v1	23	21	0	0
YOLOR-Light-v2	53	32	0	0
Mobile-YOLOR-v1	30	10	17	34
Mobile-YOLOR-v2	52	10	17	34

**Fig. 8.**
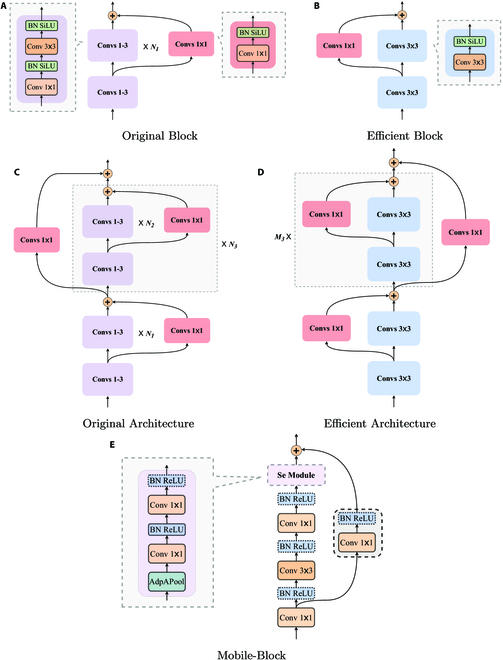
Demonstration of model lightweight strategies. In the teacher model, subfigure (A) shows an instance of its Original Block, and subfigure (C) presents the Original Architecture of the teacher model consisting of *N*_3_ Original Blocks. In the student models, subfigure (B) shows the efficient blocks obtained by simplifying the Original Blocks, and subfigure (D) shows the efficient architecture that has*M*_3_ (*M*_3_ < *N*_3_) Efficient Blocks. In particular, the Mobile-Block is derived from MobileNetv3 [45], as shown in subfigure (E), and Se Module and residual connections can be removed, and most activation functions can be replaced by H-Sigmoid.

For the first strategy, we obtain the YOLOR-Light-v1 model by simplifying the backbone and neck parts of the teacher model (YOLOR [38]). More specifically, we remove a large number of 1 × 1 convolutional layers for upscaling or downscaling in the backbone and neck parts of the teacher model and a small number of 3 × 3 convolutional layers. Looking at it from a different perspective, we propose to improve the original YOLOR model by replacing the frequently used Original Blocks with more efficient Efficient Blocks, as shown in Fig. 8A and B). We also suggest replacing the frequently occurring Original Architecture of YOLOR with more efficient Efficient Architecture, as shown in Fig. 8D. The Original Architecture is composed of Original Blocks connected by residual connections, as shown in Fig. 8C. Furthermore, we adjust the step and number of convolutional kernels in the previous layer of the deleted network layers to maintain the normal dimensionality reduction or downscaling of the features. The number of parameters of the model obtained after the above simplification operation is about 50% of the teacher model, and the Giga floating point operations (*GFLOPs*) are significantly reduced. Since the residual structure [44] has superior performance in feature extraction, we keep the residual structure in the backbone part of the model.

Another strategy is to use Mobile-Block to build the backbone of the Mobile-YOLOR-v1 model, as shown in Fig. 8E. The Mobile-Block is the block of the lightweight network MobileNetv3-Large [45] that is arranged in Mobile-YOLOR-v1 in a sequential configuration. In particular, the Se Module and residual connections can be omitted, and the activation functions of most layers can be replaced by H-Sigmoid. Moreover, the neck and head parts of the adapted YOLOR-Light-v1 model are used as its neck and head parts, thus further reducing the number of its parameters.

To make a more reasonable comparison and find a better lightweight model, we design the YOLOR-Light-v2 and Mobile-YOLOR-v2 models by slightly adding 1 × 1 and 3 × 3 convolution layers to the YOLOR-Light-v1 and Mobile-YOLOR-v1 models, but they both have a much smaller number of parameters than the teacher model. In particular, the Mobile-YOLOR-v2 model adds some additional convolutional layers from MobileNetv3-Large [45] compared to Mobile-YOLOR-v1. Similarly, YOLOR-Light-v2 has also undergone similar improvements compared to YOLOR-Light-v1. Comparative details of the performance and the number of parameters of these models, for which we report the differences between them in the result section.

#### DM

In addition to the guidance of the teacher model during the training of the student model, we use the DM to guide the student model to learn information about positive classes and bounding boxes, as shown in the detailed diagram of the detection model in Fig. 5. More specifically, we utilize *L_BCE_* to quantify the category scores difference between *L_cls_* from the real labels and *S_cls_* output by the student model. It is noteworthy that the category scores in the real labels are frequently encoded using 1-hot encoding, where the positive class has a category score of 1, and all negative classes have category scores of 0, as illustrated in the DM of Fig. 5. As a consequence, the real labels do not contain any negative class information. This is the reason why we employ a teacher model and *L_DKT_* to augment this information for the student model, as demonstrated in the head stage distiller of Fig. 5. Not only that, *L_BCE_* is also used to quantify the confidence difference between *L_obj_* from the real labels and *S_obj_* output by the student model. At the same time, we exploit *L_CIoU_* to measure the spatial information difference between *L_box_* from the real labels and *S_box_* output by the student model, which allows the model to further learn the correct spatial information. Thus, the overall loss function of the DM is as follows:

$$\mathrm{Los}s_{\mathrm{DM}}=\omega_{1}\times L_{\mathrm{BCE}}\left(S_{\mathrm{cls}},L_{\mathrm{cls}}\right)+\omega_{2}\times L_{\mathrm{BCE}}\left(S_{\mathrm{obj}},L_{\mathrm{obj}}\right)+\omega_{3}\times L_{\text{CIoU}}\left(S_{\mathrm{box}},L_{\mathrm{box}}\right),$$ (2)

where *ω*_1_, *ω*_2_, and *ω*_3_ are constants, *S_cls_*, *S_obj_*, and *S_box_* represent the category scores, the confidence, and the spatial information of the object, respectively, output from the student model, while *L_cls_*, *L_obj_*, and *L_box_* are from the real label.

### Object function

In addition to *Loss_head_* and *Loss_DM_* to provide feedback on the student model from a global perspective, we introduce *Loss_backbone_* and *Loss_neck_* to denote the loss values computed by backbone stage distiller and neck stage distiller. Thus, the final loss function of the proposed method is as follows:

$$\mathrm{Los}s_{\mathrm{distll}}=\alpha \times \mathrm{Los}s_{\mathrm{backbone}}+\beta \times \mathrm{Los}s_{\text{neck}}+\theta \times \mathrm{Los}s_{\mathrm{head}}+\gamma \times \mathrm{Los}s_{\mathrm{DM}},$$ (3)

where *α*, *β*, *θ*, and *γ* are parameters to balance the loss terms; the *Loss_backbone_* and *Loss_neck_* can be calculated by the FGD [32].

### Evaluation methods

In this subsection, to accurately evaluate the performance of the student models and the gaining effect of our method on the student models, we utilize *mAP*@ .5 and *mAP*@ .5 : .95 as the performance evaluation metric, *FPS*, *GFLOPs*, *Memory Usage*, and parameters (*Paras*) as the lightweight evaluation metric.

1. Mean of average precision at *IoU* = 0.5 (*mAP*@ .5). *mAP* is one of the most commonly used evaluation metrics in object detection, and it can comprehensively evaluate the detection effectiveness of the model for all categories of objects. That is to say, *mAP* can well reflect the multidisease detection ability of the mode, which is defined as follows:

$$\mathrm{mAP}=\frac{1}{C}\sum_{c\in \mathrm{classes}}{\mathrm{AP}}_{c}$$ (4)

where *classes* is the set of classes supported by the model, *C* is the number of categories, and *AP_c_* refers to the average precision (*AP*) for category *c*.

In the evaluation of object detection models, it is necessary to specify an intersection over union (*IoU*) threshold. A predicted bounding box is considered correct when the IoU between the predicted box and the ground-truth box exceeds or equals the specified threshold. When an *IoU* threshold of 0.5 is specified, the resulting *mAP* is denoted as *mAP*@ .5.

2. *mAP*@ .5 : .95. This is a measure of the combined performance of the object detection model at a threshold of 0.5 to 0.95 for *IoU*, which is defined as follows:

$$\mathrm{mAP}@.5:.95=\frac{1}{10}\sum_{i\in \mathrm{thresholds}}\mathrm{mAP}@.i$$ (4)

where *thresholds* = {0.5,0.55,0.60, . . . ,0.90,0.95}.

3. Frames per second (*FPS*). To measure the speed of the proposed model in processing images, we introduce the *FPS* to calculate the model efficiency.

4. Memory usage and parameters (*Paras*). Currently, the internal memory of computers is limited in reality, and large programs cannot be read into the internal memory at once or even run, so the smaller the memory usage, the easier the model can be deployed. The number of model parameters largely determines the lightness of the model and puts different pressure on the internal memory. Therefore, we introduce the parameters (*Paras*) to measure whether the proposed model is lightweight. Moreover, for the same *mAP*, the smaller number of model parameters represents a superior model.

5. Giga floating point operations (*GFLOPs*)**.** In our model, all arithmetic operations are floating-point operations, and they also directly affect the speed of the model in processing images without considering parallel operations. Even when parallel operations are considered, *GFLOPs* largely reflect the speed of the model. Therefore, we use GFLOPs to measure the complexity and lightness of our model.

## Results

### Experimental details

For the experimental settings, the implementation of the model during training and testing is based on PyTorch and runs on NVIDIA A10. The training and test datasets include 2,360 and 238 images from the PlantDoc [35] dataset, respectively. During the training stage, the model is optimized by the stochastic gradient descent optimizer with momentum set to 0.937, weight decay set to 0.0005, epochs set to 300, and batch size set to 12. We provide a set of reference values for the hyperparameters of the objective function: *α* = 1.0 × 10^−5^, *β* = 1.0 × 10^−8^, *θ* = 1.0, and *γ* = 0.5. The different student models are suitable for different hyperparameters.

To optimize the performance of the student model, we perform data cleaning on the PlantDoc [35] dataset before all training to avoid obvious errors in the labels. For example, the image size in the labels is much larger than the real image size, making the size and position of the bounding box inaccurate and possibly misleading the learning of the student models. To demonstrate the effectiveness of data cleaning, we evaluate on the student models as shown in Table 4. We find that the models perform considerably better on the data-cleaned PlantDoc [35] dataset than on the one without data cleaning. It should be noted that for fairness, none of these student models undergo knowledge distillation, and the difference in the datasets they used is only whether the labels of the training dataset are cleaned.

**Table 4.** Qualitative results of the impact of data cleaning. ↑ indicates higher is better.

Model	*mAP*@.5	*mAP*@.5
	(Uncleaned) ↑	(Cleaned) ↑
YOLOR-Light-v1	33.4	37.6
Mobile-YOLOR-v1	25.9	27.9
YOLOR-Light-v2	47.2	55.7
Mobile-YOLOR-v2	48.6	50.6

### Performance comparison

To further validate the effectiveness of the proposed student models: YOLOR-Light-v1 (distilled), Mobile-YOLOR-v1 (distilled), YOLOR-Light-v2 (distilled) and Mobile-YOLOR-v2 (distilled) models, we compare them with general image object detection methods that can be applied in plant disease analysis. These approaches include the traditional image object detection methods (Faster-rcnn-MobileNet [35] and Faster-rcnn-inception-resnet [35]) and the latest image object detection methods (YOLOv6l [46], YOLOv6m [46], YOLOv6s [46], and YOLOR [38]).

As shown in Table 5, we can find that the proposed student models outperform these image object detection methods in terms of *Paras*, *GFLOPs*, and *Memory Usage*, and even some of the metrics are far better than them, except for YOLOv6s [46]. For *mAP*@ .5, the performance of the proposed model is not much different from that of the latest image object detection methods, but far better than the traditional ones. Among them, the YOLOv6l [46] model is only 1.5% *mAP*@ .5 higher than the YOLOR-Light-v2 (Distilled) model at the expense of a huge computational cost. However, the YOLOv6s [46] model has excessive *GFLOPs* and lower *mAP*@ .5 than the YOLOR-Light-v2 model, despite its superior *Paras* and *Memory Usage*. This indicates that our proposed lightweight models utilize fewer computational resources to achieve performance comparable to that of the latest and sophisticated image object detection methods. For YOLOR-Light-v1 (distilled), Mobile-YOLOR-v1 (distilled) and Mobile-YOLOR-v2 (distilled), which require less computational resources than YOLOR-Light-v2 (distilled), they require less hardware performance, but less accuracy. A variety of different lightweight student models are proposed to cope with more application scenarios.

**Table 5.** Qualitative results of plant disease diagnosis models on the PlantDoc [35] dataset. ↑ indicates higher is better. ↓ indicates lower is better.

Model	Paras ↓	GFLOPs ↓	Memory Usage ↓	*mAP* @ .5 ↑
Faster-rcnn-MobileNet [35]	19.4M	-	74.2MB	32.8
Faster-rcnn-inception-resnet [35]	-	-	-	38.9
YOLOv6l [46]	58.5M	144.0G	112.0 MB	61.9
YOLOv6m [46]	34.3M	82.0G	71.5 MB	59.6
YOLOv6s [46]	17.2M	44.2G	36.3 MB	58.9
YOLOR [38]	37.0M	40.5G	141.6 M	60.3
YOLOR-Light-v1 (undistilled)	17.6M	23.2G	67.3 MB	37.6
Mobile-YOLOR-v1 undistilled)	17.2M	23.2G	66.1 MB	27.9
YOLOR-Light-v1 (distilled)	17.6M	23.2G	67.3 MB	42.7
Mobile-YOLOR-v1 (distilled)	17.2M	23.2G	66.1 MB	33.4
YOLOR-Light-v2 (undistilled)	20.5M	20.3G	78.4 MB	55.7
Mobile-YOLOR-v2 (undistilled)	18.2M	25.6G	72.4 MB	50.6
YOLOR-Light-v2 (distilled)	20.5M	20.3G	78.4 MB	60.4
Mobile-YOLOR-v2 (distilled)	18.2M	25.6G	72.4 MB	54.2

In order to verify the validity of the proposed MKDM for the proposed student models, we compare the models with distillation operations, as shown in Table 5. We can observe that models using the MKDM are much better than the ones without it on *mAP*@ .5. This suggests that our proposed MKDM can provide additional effective constraints on the student model and guide it to learn, resulting in higher performance.

Since many existing deep learning methods are difficult to apply in agricultural production, the main reasons are the high computational cost of existing plant disease detection methods and the difficulty of deploying the models on actual agricultural production machines. In the above comparison between the 2 aspects, it is clear that the object detection method after knowledge distillation can obtain superior plant disease diagnosis while using lower cost. Moreover, as more and more drones are put into agricultural production, efficient and accurate lightweight plant disease diagnosis models become more advantageous. This greatly promotes the development of object detection technology in agricultural production and research. In addition, plant disease diagnosis is an important part of the plant growth cycle that needs to be repeated over and over again, so the combination of a lightweight and efficient plant disease diagnosis model and an easy-to-maintain and low-cost computing device will certainly improve this part.

### Visualization analysis

**Fig. 9.**
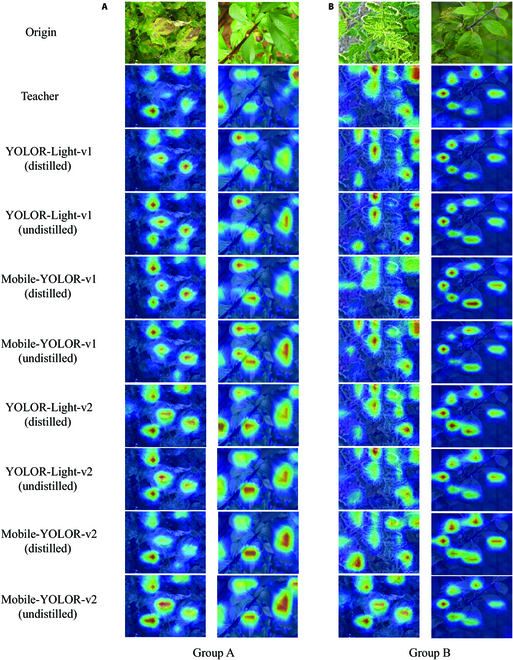
Feature visualization [47] of the last layer from models’ neck part. In this figure, warmer colors of the pixels indicate that the model assigns more attention scores to those pixels. We can observe that distillation can reduce nonkey region features (Group A) and increase key region features (Group B). (A) Group A. (B) Group B.

To visually demonstrate what enhancement can be obtained by our proposed multistage distillers, we adopt a general visualization method, Eigen-CAM [47,48], to visualize the intermediate features of the models [10]. More specifically, we extract the last layer of features in the neck of the teacher model (YOLOR [38]) and the student models (YOLOR-Light-v1, Mobile-YOLOR-v1, YOLOR-Light-v2, and Mobile-YOLOR-v2 models). Then, we utilize the Eigen-CAM [47] to plot their class activation map (CAM), through which we can visualize the model’s attention. The teacher model is capable of allocating attention more effectively to key areas, hence we can evaluate the attention allocation of student models by referring to the CAMs of the teacher model. On one hand, by referring to the CAMs of the teacher (YOLOR) model, it is evident from Group A in Fig. 9 that the student models, after multistage knowledge distillation, reduce their allocation of attention to nonkey or previously attended areas. This allows the model to better concentrate on important regions, thereby reducing misdetection and achieving more accurate localization and classification of plant diseases. On the other hand, as shown in Group B of Fig. 9, the student model can allocate attention to more key areas or increase attention allocation to existing key areas following distillation. In other words, multistage distillation can improve focus on critical regions and effectively reduce missed detection.

**Fig. 10.**
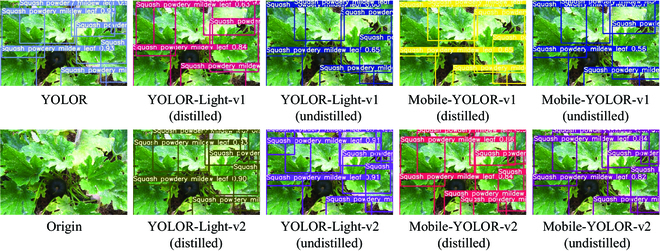
Examples of confidence enhancement after multistage distillation. The figure shows that the confidence values above the bounding box of the model output after multistage distillation has increased significantly.

**Fig. 11.**
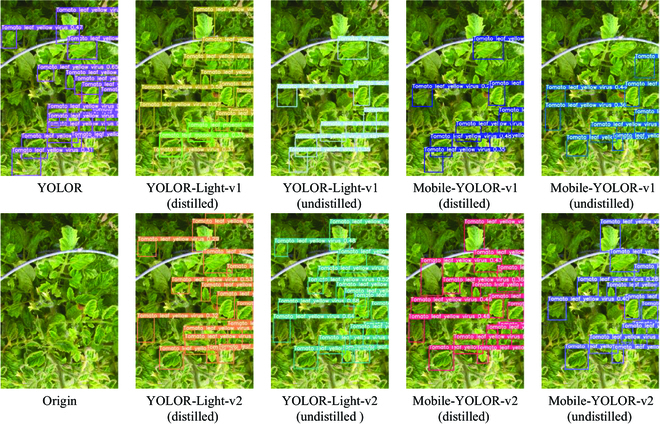
Example of improved positioning performance after multistage distillation. As can be seen from the figure, the model that has undergone multistage distillation is better able to locate the diseased leaves.

In addition, we show some output images of the teacher and student models and visualize the considerable improvement of the student models after training with the multistage knowledge distillation. More specifically, the student models after multistage knowledge distillation can significantly increase confidence in detecting the same plant disease object, as shown in Fig. 10. This means that the model became more certain about detecting the plant disease object and tended. Figure 11 shows that the distilled student models could detect more plant disease objects, although there are a few misclassification. This indicates that multistage distillation enabled the student models to significantly reduce the occurrence of missed plant disease objects.

It is worth emphasizing that having a small sample of similar categories can often result in inadequate model learning, leading to confusion between similar categories. In reality, missed plant disease detection can mislead humans to believe that the plant is healthy, missing the best time to treat the disease. It is more critical to detect more real disease objects than less misclassification of similar plant diseases.

### Ablation study

We conduct multiple experiments on student models (including YOLOR-Light-v1, Mobile-YOLOR-v1, YOLOR-Light-v2, and Mobile-YOLOR-v2 models) in the PlantDoc [35] dataset to verify the effectiveness of distillation on their backbone, neck, and head parts.

From Table 6, we can observe that the distilled student models do not necessarily perform better than the student models without knowledge distillation in terms of *FPS* (*GPU*) and *FPS* (*CPU*) but perform appreciably better in terms of *mAP*@ .5 and *mAP*@ .5 : .95, especially for the student models that use multistage distillers. These models tend to perform better in *mAP*@ .5, *mAP*@ .5 : .95, *FPS* (*GPU*), and *FPS* (*CPU*). This indicates that the student models that are trained without the guidance of the teacher model may be faster, but it is difficult to obtain excellent results in plant disease detection. The student models that use only the head stage distiller show significantly greater improvement in terms of *mAP*@ .5 and *mAP*@ .5 : .95 compared to the student models that use only the backbone or neck stage distiller. Not only that, the student models with the addition of the head stage distillation performed better on *mAP*@ .5 and *mAP*@ .5 : .95 than the one before the addition. From another perspective, when the head stage distiller is removed and only the backbone and neck stage distillers are used during training, the student model experiences a significant drop in performance in terms of *mAP*@ .5 and *mAP*@ .5 : .95. The main reason is that the head stage distiller enables the student models to effectively learn the spatial information of objects output by the teacher model to locate objects and further exploit the teacher model’s guidance to learn the diversity of plant disease categories, thus improving performance and efficiency. In addition, since the head stage distiller optimizes the student model globally, while the neck and backbone stage distillers optimize only locally, the presence of the head stage distiller often helps the student model learn better.

**Table 6.** Performance of the variations of the YOLOR-Light-v1, Mobile-YOLOR-v1, YOLOR-Light-v2, and Mobile-YOLOR-v2 models on the PlantDoc [35] dataset. √ and ✗ respectively indicate that the proposed models are distilled or not distilled at the corresponding part. ↑ indicates higher is better.

Model	Backbone	Neck	Head	*mAP*@.5 ↑	*mAP*@0.5 : .95 ↑	FPS (GPU) ↑	FPS (CPU) ↑
7*YOLOR-Light-v1	✗	✗	✗	37.6	28.1	106.4	9.5
	√	✗	✗	38.8	29.3	208.3	9.8
	✗	√	✗	38.5	28.2	270.3	9.9
	✗	✗	√	41.5	32.1	232.6	10.1
	√	√	✗	37.8	28.5	238.1	10.1
	√	✗	√	39.8	31.1	227.3	9.9
	✗	√	√	42.6	32.7	263.2	10.0
	√	√	√	42.7	32.7	217.4	10.1
7*Mobile-YOLOR-v1	✗	✗	✗	27.9	21.5	89.3	2.0
	√	✗	✗	30.5	22.4	92.6	2.1
	✗	√	✗	29.0	21.6	86.2	2.1
	✗	✗	√	33.3	23.5	88.5	2.1
	√	√	✗	30.9	22.8	92.4	2.3
	√	✗	√	32.3	23.6	94.3	2.1
	✗	√	√	33.3	23.6	92.6	2.1
	√	√	√	33.4	23.8	83.3	2.1
7*YOLOR-Light-v2	✗	✗	✗	55.7	38.7	208.3	5.8
	√	✗	✗	56.6	39.3	212.8	4.0
	✗	√	✗	56.6	39.8	227.3	3.8
	✗	✗	√	56.9	39.8	232.6	4.4
	√	√	✗	57.2	39.8	224.8	5.6
	√	✗	√	57.9	39.9	227.3	4.1
	✗	√	√	58.1	40.0	232.6	4.8
	√	√	√	60.4	41.2	256.4	3.6
7*Mobile-YOLOR-v2	✗	✗	✗	50.6	35.0	105.3	1.8
	√	✗	✗	51.0	35.4	103.1	1.8
	✗	√	✗	52.4	35.9	105.3	1.9
	✗	✗	√	52.4	36.3	106.4	2.0
	√	√	✗	53.0	36.5	106.7	2.3
	√	✗	√	53.4	37.2	107.5	1.8
	✗	√	√	52.4	36.8	104.2	1.9
	√	√	√	54.2	37.4	115.0	3.4

In summary, backbone, neck, and head stage distillers can not only individually provide beneficial enhancements to student models but also achieve better gains when working together.

### Evaluation model lightweighting

The lightness of a model largely determines how easily it can be deployed in real-world agricultural disease application scenarios. The degree of lightness is measured in terms of *Paras*, *GFLOPs*, *MemoryUsage*, and inference speed on GPU and CPU. Table 7 reveals that the YOLOR-Light-v2 model surpasses the YOLOR [38] model in terms of *Paras*, *GFLOPs*, *MemoryUsage*, *FPS* (*GPU*), and *mAP*@ .5, except for *FPS* (*CPU*). YOLOR-Light-v1 and Mobile-YOLOR-v1 exhibit lower *Paras*, *GFLOPs*, and *MemoryUsage* compared to YOLOR-Light-v2 and Mobile-YOLOR-v2, indicating that they require less computational resources. YOLOR-Light-v1 achieves the highest *FPS* (*GPU*) and *FPS* (*CPU*) among all 4 student models, indicating its superior speed compared to the other models. It should be noted that *FPS* (*GPU*) and *FPS* (*CPU*) are influenced by various factors, including the network inference time of the model network (backbone, neck, and head) and the time taken by NMS to filter bounding boxes. While optimizing the network structure may reduce inference time, it may also affect the accuracy and compromise the quality of the raw input given to NMS, thereby necessitating more execution time for the NMS algorithm. YOLOR-Light-v2 achieves the highest *mAP*@ .5, indicating its superior detection accuracy, and considering other metrics, it strikes the best balance between lightweight and accuracy. The results demonstrate that employing multistage distillation techniques during training enables lightweight student models to attain the performance levels of the teacher model YOLOR [38], rendering them highly suitable for practical agricultural applications. However, the performance of the further lightened Mobile-YOLOR-v2 model is not as good as that of YOLOR-Light-v2, and there is a significant decrease in the performance of the YOLOR-Light-v1 and Mobile-YOLOR-v1 models. This suggests that pursuing model lightweighting too much may crash the model performance. In addition, it can be seen from Table 7 that our student models are comparable to the previous lightweight model, Faster-rcnn-MobileNet, in terms of *Paras* and *MemoryUsage* while achieving higher accuracy, and have a greater advantage over the cutting-edge YOLOv6 [46] models in terms of lightness. This means that our student model requires fewer computer hardware and software resources to perform plant disease diagnosis tasks. Moreover, with the reduced requirements for computer hardware and software resources, there are more computing devices available.

**Table 7.** Details of the proposed student model compared with the teacher model and its deformations in terms of lightness. ↑ indicates higher is better. ↓ indicates lower is better.

Model	Paras ↓	GFLOPs ↓	Memory Usage ↓	FPS (GPU) ↑	FPS (CPU) ↑	*mAP*@0.5 ↑
YOLOR [38]	37.0M	40.5G	141.6 MB	175.4	5.2	60.3
YOLOR-Light-v1	17.6M	16.6G	67.3 MB	263.2	10.1	42.7
Mobile-YOLOR-v1	17.2M	23.2G	66.1 MB	94.3	2.1	33.4
YOLOR-Light-v2	20.5M	20.3G	78.4 MB	256.4	3.6	60.4
Mobile-YOLOR-v2	18.2M	25.6G	72.4 MB	115.0	3.4	54.2

As more and more computing devices are equipped with small GPUs, the lighter student models we proposed can be more easily deployed and used for real-world agricultural applications. Plant disease diagnosis is a process that requires constant repetition and has high real-time requirements. Therefore, the combination of low-use and low-maintenance computing devices and lightweight plant disease diagnostic models has great potential in agricultural production and will be favored by agricultural producers [49].

### Impact of the initial values of the object boxes

The initial width and height of bounding boxes output by the model are important for plant disease diagnosis methods based on object detection. If the values are reasonable, the object bounding boxes generated by the model will be close to the ground truth. We apply the 12 pairs of width and height values from Table 2 to the head parts of the teacher and student models as the initial width and height values for their output bounding boxes. To fairly demonstrate the impact of object box initialization on the proposed model, we have trained 4 student models using width and height initial values obtained from the COCO dataset and compare them with student models using initial values from Table 2, without distillation. For the sake of fairness, none of these models have been distilled.

In detail, Table 8 shows that the student models consistently achieved higher *mAP* at the same *IoU* threshold when using initial width and height values obtained from Table 2. This suggests that the initial values extracted from the PlantDoc [35] dataset are more appropriate for plant disease detection.

**Table 8.** Qualitative results of the impact of the initial values of object boxes. ↑ indicates higher is better.

Model	Source of boxes	*mAP* @ .5 ↑	*mAP* @ .65 ↑	*mAP* @ .80 ↑	mAP@0.5: .95 ↑
2*YOLOR-Light-v1	COCO [37]	36.8	36.7	36.3	27.6
	PlantDoc [35]	37.6	37.2	36.4	28.1
2*YOLOR-Light-v2	COCO [37]	55.3	55.2	54.4	38.4
	PlantDoc [35]	55.7	55.5	54.7	38.7
2*Mobile-YOLOR-v1	COCO [37]	26.9	26.8	26.0	20.2
	PlantDoc [35]	27.9	28.5	27.7	21.5
2*Mobile-YOLOR-v2	COCO [37]	48.0	47.9	46.9	32.3
	PlantDoc [35]	50.6	50.4	49.3	35.0

This indicates that knowledge obtained from a specific plant disease dataset is more valid than that obtained from a common dataset in the object detection field. This knowledge enables the model to locate the lesions of plant diseases more precisely, and the precise localization can effectively advance the quantification of plant diseases based on object detection.

## Discussion

Our proposed multistage distillation can effectively improve the performance of the student models for plant disease detection, and the student models are more lightweight, which makes their deployment and application easy. In agricultural production environments, computing power is often scarce, and some advanced equipment is unsuitable for working in agricultural fields because of its harsh use and maintenance conditions. The lightweight models with low equipment requirements and maintenance costs will receive increasing attention and application in such environments.

Moreover, through our work, we have identified some key issues that still exist: fine-grained recognition of plant diseases, dense and small objects detection, and data annotation problem. We discuss these issues and trends in different sections.

### Fine-grained recognition

**Fig. 12.**
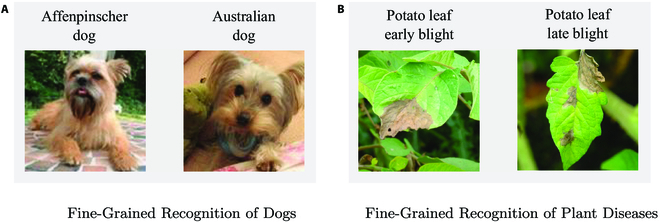
Examples of fine-grained recognition. The images of the dog are from Stanford Dogs Dataset [54], and the images of the potato are from PlantDonc [35] Dataset. (A) Fine-grained recognition of dogs. (B) Fine-grained recognition of plant diseases.

Fine-grained recognition [50–52] is a research focus in computer vision, and plant disease recognition also faces similar challenges as fine-grained recognition. Fine-grained recognition refers to accurately classifying each subcategory within a large category with multiple subcategories by identifying small but important differences. In fine-grained recognition tasks, the differences between different sub-categories are usually small, and these differences can only be distinguished through careful observation and analysis. The application scope of fine-grained recognition is wide, including animal species classification, flower species identification, and vehicle model recognition, as shown in Fig. 12A. For plant disease recognition, there are many categories of plant diseases, and sometimes there are only small differences between different diseases, as shown in Fig. 12B. In addition, the fine-grained recognition of plant diseases [53] is also influenced by environmental and growth factors. Future research in plant disease diagnosis should focus on fine-grained disease identification in multiple crops. Since the characteristics of most plant diseases vary from period to period, we suggest classifying them for different disease periods in order to study subsequent disease treatment efforts. What kind of plant disease subcategory and standard is the most effective, which is also worthy of our exploration.

Moreover, fine-grained recognition emphasizes interclass differences, and our proposed *L_DKT_* has a strong migration capability for class diversity. Therefore, our proposed multistage distillation possesses the potential to be applied on plant disease fine-grained recognition.

### The detection of dense and small objects

Most plant diseases have small lesions, especially when photographs are taken with drones and cameras mounted at high and distant locations, where the lesion area is minimal compared to the background. In this case, it poses a challenge for object detection-based plant disease diagnosis models. Especially for CNN-based models, after multiple convolutions, the details of the original image become blurred, and eventually, the information on small objects becomes blurred or even lost. Moreover, the lesions are even less visible in the early stages of plant disease development. Improving the model’s performance for small object detection will inevitably enable the accurate detection of plant diseases at the early stage of their occurrence and effectively suppress their spread, ultimately leading to savings in agricultural production expenses and yield improvement.

Due to the dense and overlapping nature of some plant diseases, traditional object detection algorithms may not accurately identify each lesion, making it difficult to count them. Therefore, specialized dense object detection algorithms are required. Additionally, some tasks, such as wheat counting, inherently involve the operation on dense objects. Besides, since most current object detection relies on NMS algorithms to remove duplicate objects, this point results in many densely distributed objects that need to be correctly filtered out. This is detrimental to counting disease lesions and estimating disease severity, making it necessary to explore alternative algorithms to NMS. Applying dense object detection to the detection and counting of plant diseases at the lesion level can facilitate the process of fine plant disease diagnosis.

It is worth mentioning that both dense object detection and small object detection have remarkable similarities with traditional object detection. Therefore, the multistage distillation method proposed by us for object detection is also feasible for application in dense and small object detection.

### Multipurpose images and annotations

The available plant disease images only cover some factual situations, and some situations may occur less frequently, for example, crops infected with multiple diseases simultaneously. Only 0.74% of the images in the PlantDoc [35] contain multiple diseases. Some situations occur very infrequently, and the economic and labor costs of photographing and labeling images are high. However, as the value of the data increases, the investment cost will be worth it.

**Fig. 13.**
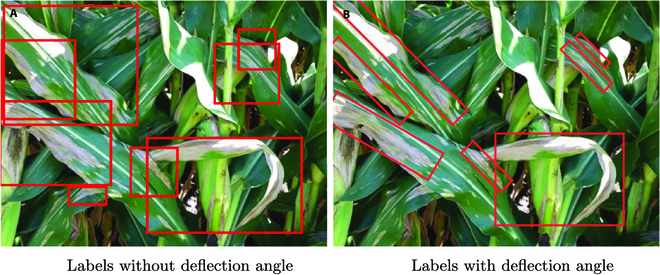
More accurate labeling information. (A) Labels without deflection angle. (B) Labels with deflection angle.

In addition, correct and effective labels are even more scarce than plant disease images. Some plant disease detection datasets are annotated with disease categories, and these datasets can only be used for classification tasks. Some datasets use bounding boxes as annotations and give category information, which can effectively describe the area and location of most lesions. However, this format is unsuitable for strip lesions, and the bounding boxes cover too much non-lesioned area, as shown in Fig. 13A. Providing multiple annotations for a set of images is complex and laborious, and the operational and labor costs required for different annotation information are different. Therefore, we propose manually providing only annotations for the instance segmentation and then designing algorithms to convert these annotations into annotation information for classification or detection. In order to describe the plant lesions more precisely, we propose adding deflection angle information to the annotation of each object’s bounding box, as shown in Fig. 13B.

## Data Availability

We released our code and data at https://github.com/QDH/MSKD.
